# “First” abyssal record of *Stenosemus exaratus* (G.O. Sars, 1878) (Mollusca, Polyplacophora) in the North-Atlantic Ocean

**DOI:** 10.3897/zookeys.283.4704

**Published:** 2013-04-03

**Authors:** Louise Allcock, Enrico Schwabe

**Affiliations:** 1 Ryan Institute and School of Natural Sciences, National University of Ireland Galway, University Road, Galway, Ireland; 2 Bavarian State Collection of Zoology, Münchhausenstr. 21, 81247 München, Germany

**Keywords:** Deep-sea, Irish Sea, canyon systems

## Abstract

The first proven abyssal record of *Stenosemus exaratus* (G.O. Sars, 1878) is presented on the basis of an ROV study in the Irish Sea. For the first time *in situ* images of the species and data on the environmental parameters are provided.

## Introduction

Polyplacophoran molluscs are a group of exclusively benthic organisms distributed worldwide that are found from the splash zone down to hadal depths (Schwabe 2008).

According to Schwabe (2008) the maximum depth in which *Stenosemus exaratus* (G.O. Sars, 1878) has been collected is 2580 m. Schwabe (2008) cited depth ranges for this species cited by Kaas and Van Belle (1990), but confirmed localities where the species was collected in abyssal depths could not be traced. Thus proof for the occurrence of *Stenosemus exaratus* below the continental rise *sensu* Gage and Tyler (1991) is still lacking. The only abyssal records of chitons in the North Atlantic (excluding the Caribbean Sea) are restricted to a handful of records from off Galicia and the Bay of Biscay and all refer to *Leptochiton alveolus* (M. Sars MS, Lovén, 1846). Kaas and Van Belle (1994) were apparently also aware of abyssal records for *Placiphorella atlantica* (Verrill & S. I. Smith in Verrill, 1882), but subsequent research again failed to trace these (Schwabe 2008). Thus *Leptochiton alveolus* is so far the only “true” abyssal Northern Atlantic species for which precise occurrence records are available.

During an expedition exploring canyon systems to the southeast of the Rockall Trough on the shelf edge of Ireland, one of us (LA) was able to collect three specimens of *Stenosemus exaratus* by means of an ROV (remotely operated vehicle). Still and high-definition video camera systems provide for the first time an insight into the species’ habitat. In addition, data are presented on relevant environmental parameters.

## Material and methods

The chitons recorded here were collected during survey CE10004 of RV Celtic Explorer. This cruise, entitled ‘*Species at the Margins*’ sampled an unnamed canyon system at the edge of the continental margin, north of the Porcupine Bank, using the Irish deep-water ROV Holland I. ROV Holland I is a Quasar work class ROV rated to 3000 m. It is equipped with several video camera systems including a Kongsberg OE14-502a high definition colour zoom and a Kongsberg OE14-208 digital stills camera, and has two robotic arms and a slurp sampler. Laser sights are positioned 10 cm apart to facilitate size estimates. Samples from the slurp sampler are maintained in an enclosed system for the duration of the dive. Fauna collected with the robotic arms are stored in extendable storage boxes. Once samples arrived on deck, they were hand-picked from the ROV boxes and sediment was sieved through a 500 μm mesh. The chitons were deposited and identified at the Bavarian State collection of Zoology (ZSM Mol 20110215) (by ES). Environmental parameters were obtained using a 24-rosette conductivity-temperature-depth (CTD) data logger from the nearest locality and by visual inspection of the sediment. According to the available video sequences the species was collected at 2:06 pm. The position of the ROV was determined using a global acoustic positioning system, which incorporates inertial navigation systems and global positioning using ultra-short baseline beacons. At these depths, position data can be intermittent. We obtained position data approximately 10 minutes after the chiton was collected. As the ROV was climbing a vertical wall during this period, only the depth value is slightly inaccurate, the actual collection depth being slightly (approximately 20 meters as estimated from video footage) deeper than the nearest datum point.

## Data resources

The data underpinning the analysis reported in this paper are deposited in the Dryad Data Repository at doi: 10.5061/dryad.h261h.

## Results

**Figure 1.**
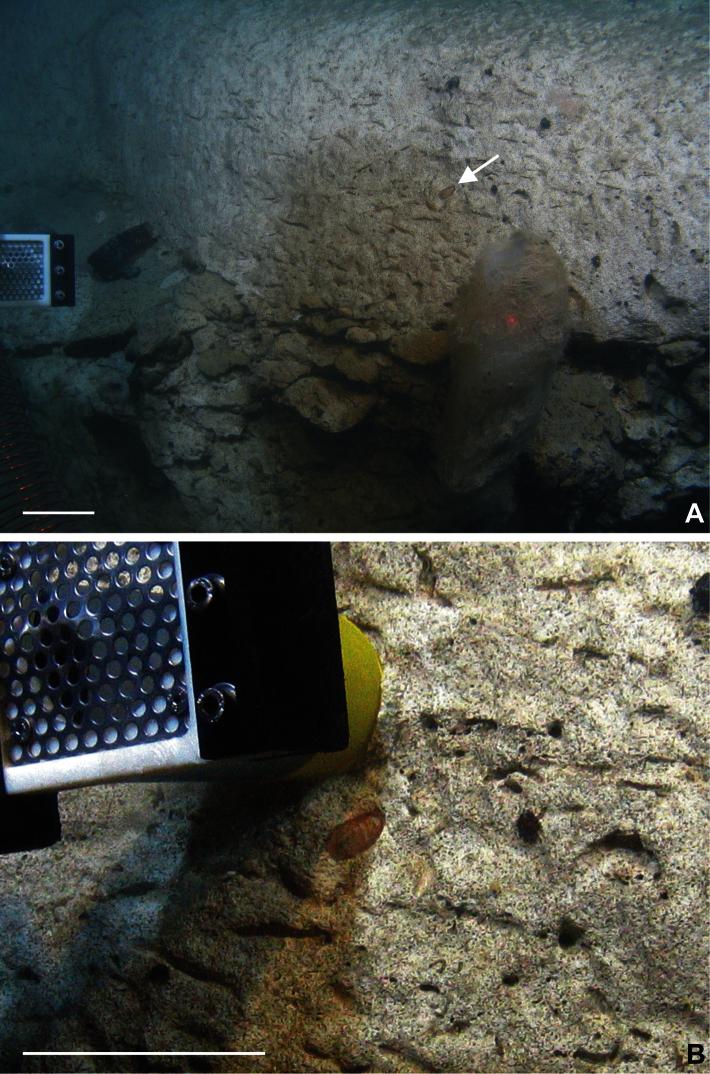
Rockall Trough at station 96 at 54.2172°N, 12.6598°W in 2733 m. The camera system of ROV “Holland I” detects *Stenosemus exaratus* (G.O. Sars, 1878) in its natural environment for the first time. A - Macrofauna-poor impression of the steep canyon wall; foreground contains a 30 cm long glass sponge and the chiton (indicated by an arrow). B - The ROV slurp sampler attempts to remove the chiton. Scale bars 10 cm.

During station 96 of cruise CE1004, an ROV dive to 3000 m depth, three full grown specimens of *Stenosemus exaratus* (G.O. Sars, 1878) were collected on a steep wall of an unnamed canyon southeast of the Rockall Trough (Fig. 1) at 54.2172°N, 12.6598°W. One specimen was sighted and taken just below the nearest recorded depth of 2733 m. Two additional specimens were taken blind by the slurp sampler, from wall sediment during the course of the dive. The wall extends vertically from approximately 2800 to 2650 m and consists of chalk, but is covered all over by a very fine greenish-grey silt layer. Despite a remarkably high number of scars and micro cavities the only other obvious macrobenthic fauna close to the sighted chiton was a glass sponge approximately 30 cm in length. No feeding tracks or “home” marks were visible around the chiton.

Data from the CTD at station 93 (54.217°N, 12.661°W, depth 2733 m) reveals the following abiotic parameters: salinity 34.925, temperature 2.85°C, pressure 2775.87 db and oxygen 235 µmol/kg (this corresponds to a saturation of about 72–73%). These data indicate that the Bay of Biscay area is influenced by cold oxygen-rich Labrador Sea Water (e.g., McGrath et al. 2012)

## Discussion

**Figure 2.**
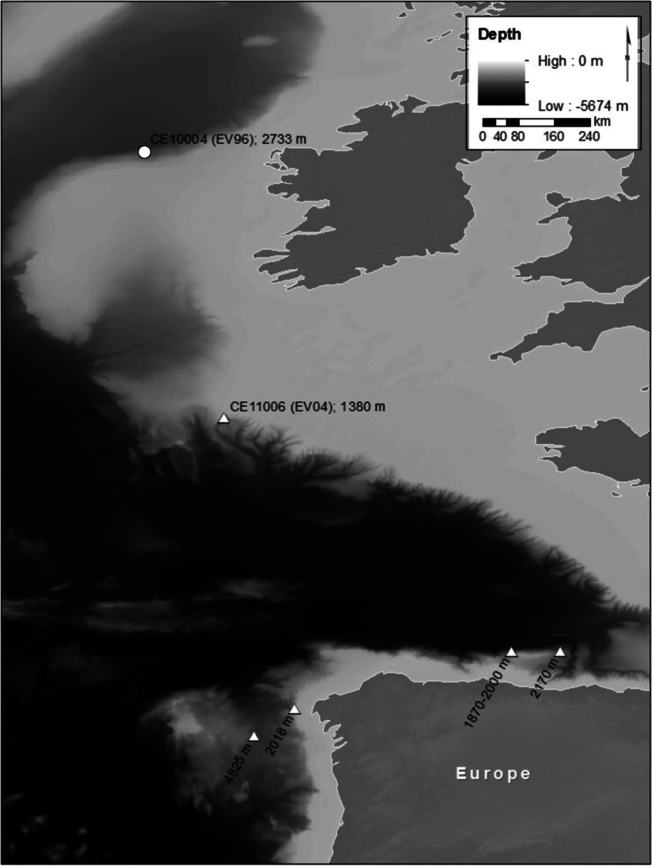
Locality map for the deep-water records of *Stenosemus exaratus* (G.O. Sars, 1878) (circle, herein) and *Leptochiton alveolus* (M. Sars MS, Lovén, 1846) (triangles) in the North Atlantic. “CE” localities refer to our expeditions on board the RV *Celtic Explorer*, remaining data extracted from Schwabe (2008).

Abyssal records of chitons are scarce and few species are known to inhabit depths below the continental slope (see Schwabe 2008). Schwabe (2008) also showed that eurybathy occurs very rarely in polyplacophorans. Among the few species exhibiting eurybathy is *Stenosemus exaratus* reported herein. The present finding represents its deepest record (Fig. 2, circle), but it also occurs rather shallowly in fjord systems, including the Chilean Fjord region, where Schwabe and Sellanes (2010) recorded the shallowest occurrence at 23 m.

A similar situation was revealed for the other North Atlantic abyssal species, *Leptochiton alveolus* (Fig. 2, triangles). While its abyssal records to date are restricted to the canyon regions of the Bay of Biscay, we found it at 1380 m during cruise CE11006 of RV Celtic Explorer during a dive of ROV Holland I under a *Lophelia pertusa* bank in the Whittard Canyon. This coral species was also recorded at 1350 m in the Whittard Canyon system by Huvenne et al. (2011).

Mortensen and Fossa (2006, as *Lepidochitona* [sic] *alveolus*) reported *Lepidochitona alveolus* from living cold-water coral *Lophelia pertusa* reefs in the Midfjord (Norway) in depths between 150–160 m. The previous deep-water findings of *Leptochiton alveolus* in the Bay of Biscay (Fig. 2) region lack accompanying data and it remains unclear, if the species is somehow related to the occurrence of *Lophelia pertusa*. However, hypothetically this would be possible, as Davies and Guinotte (2011: figs 4, 5) demonstrated that a co-occurrence of both species is possible. Jensen and Frederiksen (1992), however, did not record a single chiton from *Lophelia* associated communities.
